# Aneurysm, Pseudoaneurysm, Diverticulum, or Other? Discordance Between Multimodality Imaging and Surgical Findings in a Patient with Coronary Artery Disease

**DOI:** 10.3390/life16060908

**Published:** 2026-05-28

**Authors:** Iulia Raluca Munteanu, Ramona Cristina Novaconi, Adrian Grigore Merce, Daniel Nica-Dalia, Horea Bogdan Feier

**Affiliations:** 1 Doctoral School Medicine-Pharmacy, “Victor Babes” University of Medicine and Pharmacy Timisoara, E. Murgu Sq. No. 2, 300041 Timisoara, Romania; iulia.munteanu@umft.ro (I.R.M.); adrian-grigore.merce@umft.ro (A.G.M.); 2 Advanced Research Center, Institute for Cardiovascular Diseases, 300310 Timisoara, Romania; horea.feier@umft.ro; 3 Institute for Cardiovascular Diseases of Timisoara, Clinic of Cardiovascular Surgery, Gheorghe Adam Street, No. 13A, 300310 Timisoara, Romania; dnicad@yahoo.com; 4 Department VI Cardiology, “Victor Babes” University of Medicine and Pharmacy Timisoara, E. Murgu Sq. No. 2, 300041 Timisoara, Romania

**Keywords:** left ventricular aneurysm, left ventricular pseudoaneurysm, multimodality imaging, case report, coronary artery bypass grafting, diagnostic discordance

## Abstract

Background: Left ventricular outpouchings remain among the most difficult structural abnormalities to classify in clinical practice. The differential diagnosis usually includes true aneurysm, pseudoaneurysm, diverticulum, and less clearly defined chronic post-ischemic remodeling patterns. Although multimodality imaging is central to preoperative assessment, it may still overstate diagnostic certainty in complex chronic lesions. Case Presentation: We report the case of a 66-year-old man with chronic coronary syndrome and severe multivessel coronary artery disease in whom transthoracic echocardiography, computed tomography, ventriculography, and cardiac magnetic resonance consistently suggested a basal lateral left ventricular pseudoaneurysm, with imaging findings compatible with an associated mural thrombotic component. Because of the coexistence of surgically significant coronary disease, the patient was referred for operative treatment. Intraoperatively, however, the expected pseudoaneurysmal cavity was not identified. Instead, two posterolateral fibro-calcific left ventricular formations were found in a surgically difficult area, with an appearance that did not correlate convincingly with any preoperative imaging study. Given their calcified aspect, difficult exposure, and the high risk of additional surgical manipulation, no direct intervention was performed on these structures, and only myocardial revascularization was undertaken. The postoperative course was favorable. Discussion: The case highlights a clinically important limitation of multimodality imaging: concordant imaging does not necessarily equal an anatomically correct diagnosis. The discrepancy between imaging and operative findings raises unresolved questions as to whether the lesion represented small chronic aneurysmal formations, an unusual chronic pseudoaneurysm, a calcified diverticular process, multiple fibro-calcific post-infarction outpouchings, or another form of chronic left ventricular remodeling. Rather than forcing a definitive label unsupported by pathology, the case is better understood as a diagnostic gray-zone lesion. Conclusions: Even comprehensive imaging may remain incomplete when evaluating unusual left ventricular outpouchings in ischemic patients. This case underscores the need for cautious diagnostic language, close correlation with operative findings, and broader discussion regarding the classification of chronic left ventricular parietal lesions.

## 1. Introduction

Left ventricular outpouchings are uncommon but clinically consequential findings, particularly in patients with ischemic heart disease. Their differential diagnosis includes true aneurysm, pseudoaneurysm, congenital or acquired diverticulum, contained post-infarction rupture, and other less clearly defined forms of chronic ventricular wall remodeling [1,2,3]. Distinguishing among these entities matters because the associated risk profile, surgical implications, and prognosis differ substantially [4].

Among them, left ventricular pseudoaneurysm is generally regarded as the most critical diagnosis, as it reflects contained myocardial free-wall rupture and may carry a risk of rupture [1,3]. By contrast, ventricular diverticula are classically described as outpouchings containing all myocardial layers and are most often considered congenital. In practice, however, this distinction is not always straightforward, especially in ischemic patients with scar, mural thrombus, calcification, and distorted ventricular geometry. In such patients, mural thrombus may coexist with ventricular outpouchings, ischemic scar, or chronic remodeling and may contribute to diagnostic uncertainty. Moreover, thrombotic material may occasionally mimic or obscure other intracardiac masses, making standardized echocardiographic assessment and careful multimodality correlation essential [5].

Modern cardiovascular imaging has markedly improved lesion detection and characterization. Echocardiography, computed tomography, ventriculography, and cardiac magnetic resonance can provide complementary information regarding neck morphology, wall continuity, thrombus, fibrosis, and regional mechanics [6,7,8,9]. Nevertheless, even when these modalities appear concordant, interpretation may remain inferential rather than definitive in atypical chronic lesions [8].

We report the case of a patient with severe multivessel coronary artery disease in whom multimodality imaging repeatedly suggested a left ventricular pseudoaneurysm, whereas surgery revealed two calcified posterolateral formations that did not match the expected anatomy. The case is presented as a diagnostic dilemma rather than a resolved entity, with particular emphasis on the limits of imaging-based certainty in complex left ventricular lesions.

## 2. Case Presentation

A 66-year-old man with chronic coronary syndrome was referred for surgical evaluation in the setting of multivessel coronary artery disease and a suspected structural lesion of the left ventricle. His medical history included hypertension, mixed dyslipidemia, chronic smoking, chronic kidney disease, overweight status, and heart failure (New York Heart Association class II). Initial cardiology assessment had already documented severe coronary artery disease involving the right coronary artery, left anterior descending artery, and obtuse marginal branches, together with a basal lateral left ventricular outpouching considered highly suggestive of pseudoaneurysm.

The patient reported several months of chest pain, predominantly effort-related but occasionally present at rest, associated with exertional dyspnea and fatigability.

Electrocardiography showed sinus rhythm at 60 bpm, intermediate QRS axis, and negative T waves in the lateral leads, without acute ischemic changes.

Transthoracic echocardiography showed a non-dilated left ventricle with an ejection fraction of approximately 50%, regional wall motion abnormalities involving the posterior, inferior, and lateral walls, and a basal anterolateral/lateral left ventricular outpouching initially interpreted as compatible with pseudoaneurysm. On retrospective review, the echocardiographic image could also be interpreted as showing two adjacent outpouching-like spaces, separated by a hyperechogenic structure possibly corresponding to calcification. The superior cavity was measured at approximately 18.3 × 18.6 mm, whereas the inferior adjacent space, located below the posterior mitral leaflet, was not separately measured at the time of the initial examination. No definite mural thrombus or intracavitary mass could be confidently characterized on standard transthoracic echocardiography. The echocardiographic assessment focused on the morphology of the outpouching, its apparent communication with the left ventricular cavity, regional wall motion abnormalities, and its relationship with the adjacent ventricular wall. Contrast-enhanced echocardiography and transesophageal echocardiography were considered as possible complementary techniques; however, cardiac magnetic resonance was prioritized because of its ability to provide tissue characterization and assess scar, wall continuity, and thrombotic components in the same examination (Figure 1).

Coronary angiography demonstrated a right-dominant coronary system with chronic occlusion of the right coronary artery, proximal occlusion of the first obtuse marginal branch, proximal subtotal occlusion of the second obtuse marginal branch (OM), and a long 75–90% stenosis in the mid left anterior descending artery (LAD). Left ventriculography showed contrast opacification of a lateral wall outpouching-like cavity, measuring approximately 2.0 × 2.5 cm, without contrast extravasation into the pericardial space. At the time of the initial interpretation, this was described as a single communicating cavity. However, in light of the intraoperative finding and retrospective review of the echocardiographic and 3D CT images, the possibility that ventriculography opacified two adjacent or partially overlapping pouch-like structures cannot be excluded. The original ventriculographic image was not available for inclusion, and this has been acknowledged as a limitation.

Contrast-enhanced computed tomography demonstrated a well-defined left ventricular lateral wall outpouching-like abnormality with homogeneous opacification synchronous with the left ventricular cavity, indicating communication with the ventricular lumen. On the standard axial section, the abnormality appeared as a single dominant cavity and was initially interpreted as compatible with an aneurysmal/pseudoaneurysmal lesion. However, retrospective assessment of the three-dimensional volume-rendered reconstruction suggested a more complex morphology, with two adjacent or partially overlapping pouch-like protrusions. The available standard CT planes were not sufficient to reliably define the individual orifice of each suspected pouch (Figure 2).

Additional review of the available preoperative CT dataset, including coronal and oblique reconstructions, suggested a more complex morphology than initially appreciated on standard axial sections. These supplementary views showed a basal/lateral left ventricular outpouching-like abnormality with possible adjacent or partially overlapping pouch-like components, with a maximal measured dimension of approximately 3.08 cm. However, the available CT dataset did not allow definitive separation of the individual orifice of each suspected component (Supplementary Figure S1).

At preoperative reassessment in the cardiovascular surgery department, the patient remained hemodynamically stable and free of angina at rest. Repeat transthoracic echocardiography showed no changes.

Cardiac magnetic resonance further reinforced the pseudoaneurysm hypothesis. Cine imaging showed a normal-sized left ventricle with akinesia of the medio-basal lateral wall and focal basal discontinuity associated with a dominant 20 × 23 mm outpouching, with a narrow neck of approximately 7 mm. Only one dominant orifice was clearly identified on the available standard CMR planes. In retrospect, and in correlation with echocardiography, 3D CT reconstruction, and intraoperative findings, the possibility of a second adjacent pouch-like component cannot be excluded. Dedicated off-axis cine and late gadolinium enhancement planes targeted to the suspected lesion might have better separated the two components and clarified their relationship with the ventricular wall. Global systolic function appeared moderately reduced when the lesion was included in the volumetric analysis, with an ejection fraction of 39% and end-systolic volume of 68 mL, but was preserved when excluded, with an ejection fraction of 60% and end-systolic volume of 44 mL. T2-weighted imaging showed no myocardial edema. Native T1 mapping values were globally normal. Early gadolinium enhancement suggested a possible mural thrombotic component, while late gadolinium enhancement showed transmural fibrosis in the medio-basal lateral wall with an overlying hypointense component compatible with thrombus. Additional subendocardial delayed enhancement with mural extension was present in the inferior and inferoseptal walls, with residual viability estimated at 50–75% in that territory. The imaging conclusion was old lateral and inferior myocardial infarction with findings compatible with a left ventricular lateral wall pseudoaneurysm and suspected associated mural thrombus (Figure 3).

Taken together, the preoperative studies strongly favoured a chronic left ventricular pseudoaneurysm.

Double coronary artery bypass grafting (CABG) was carried out (left internal mammary artery anastomosed to the left anterior descending artery and an autologous saphenous vein graft to the first obtuse marginal branch). Intraoperatively, however, the suspected pseudoaneurysm/diverticulum did not correspond to the preoperative expectation. Rather than a single, clearly defined pseudoaneurysmal cavity, two small fibro-calcific outpouching-like formations were identified on the posterolateral aspect of the left ventricle in a surgically difficult-to-access area. Their external wall appeared grossly continuous with the ventricular wall. This feature made small chronic aneurysmal formations a plausible alternative interpretation, possibly more likely than a classic pseudoaneurysm, although no definitive classification was possible in the absence of resection or histopathology. Because of difficult exposure, the chronic calcified appearance of the lesions, and the high incremental risk of further intervention, no direct surgical maneuver was performed on them. The procedure was therefore limited to myocardial revascularization. This intraoperative discrepancy became the defining feature of the case (Figure 4).

The postoperative course was overall favourable. Follow-up echocardiography showed a non-dilated left ventricle with inferobasal akinesia, an ejection fraction of approximately 50%, mild mitral regurgitation, mild functional tricuspid regurgitation, and no pericardial or pleural effusion. The patient was discharged in good condition. The main clinical events and imaging findings are summarized in Table 1. Notably, the surgical discharge summary retained the diagnosis of left ventricular pseudoaneurysm but also referred to medio-basal lateral wall fibro-calcific parietal diverticula, reflecting the unresolved interpretive shift introduced by the intraoperative findings. Postoperatively, given the absence of intraoperative confirmation of a free thrombotic mass and the lack of pathological diagnosis, anticoagulation was not initiated routinely solely on the basis of the preoperative imaging suspicion. Antithrombotic management was individualized according to the patient’s coronary revascularization, bleeding risk, and overall clinical status. Follow-up transthoracic echocardiography was planned at 6 months to reassess left ventricular function, the persistence or evolution of the lateral wall lesion, and any residual imaging findings compatible with thrombus.

## 3. Discussion

The central value of this case lies in the mismatch between diagnostic confidence and operative anatomy [4]. All major preoperative imaging modalities converged on a single working diagnosis: chronic left ventricular pseudoaneurysm [6,9]. Yet surgery did not confirm the expected lesion morphology. Instead, it revealed two chronic fibro-calcific formations in a posterolateral location, without a convincing one-to-one anatomical correspondence with the preoperative studies. Retrospective correlation of echocardiography, 3D CT reconstruction, and intraoperative inspection suggests that the lesion may have been more complex than initially appreciated, possibly consisting of two adjacent or partially overlapping pouch-like structures rather than a single cavity.

This matters because, in structural heart disease, concordance between multiple imaging modalities is often taken as a surrogate for diagnostic certainty. Our case cautions against that assumption. Multimodality imaging may increase confidence, but it does not eliminate interpretive error, particularly in chronic ischemic lesions where scar, mural thrombus, calcification, and geometric distortion coexist [6,7].

### 3.1. Why Pseudoaneurysm Was a Reasonable Preoperative Diagnosis

The pseudoaneurysm hypothesis was not weak or careless; on the contrary, it was strongly supported. Computed tomography described a basal lateral pseudoaneurysm. Echocardiography repeatedly identified a lateral outpouching with abnormal wall motion. Ventriculography demonstrated a cavity communicating with the left ventricle. Cardiac magnetic resonance added the most compelling features: a narrow neck, apparent wall discontinuity, suspected mural thrombotic component, and adjacent transmural scar in a patient with prior infarction. Taken together, these findings were entirely compatible with a chronic pseudoaneurysm.

### 3.2. Single Versus Double Outpouching Morphology and the Limits of Standard Imaging Planes

A key point raised by retrospective image–surgery correlation is whether the lesion was truly a single outpouching or whether two adjacent pouch-like structures were present from the beginning but incompletely characterized by standard imaging planes. On transthoracic echocardiography, the measured cavity was interpreted as a single lateral wall outpouching. However, retrospective review suggests a possible second inferior pouch-like space below the posterior mitral leaflet, separated from the measured component by a hyperechogenic structure, possibly calcific. Similarly, the standard axial CT section and the displayed CMR section predominantly showed one dominant cavity, whereas the 3D CT reconstruction and the intraoperative image suggested a more complex morphology with two adjacent protrusions.

This discrepancy highlights an important practical limitation of standard imaging protocols. Conventional echocardiographic, CT, and CMR planes may be sufficient for common ventricular aneurysms or pseudoaneurysms, but they may fail to separate adjacent or partially overlapping cavities in atypical chronic post-ischemic lesions. In such cases, imaging should be adapted to the specific anatomy rather than limited to standard planes. Lesion-targeted off-axis views, dedicated cine and late gadolinium enhancement CMR planes, multiplanar CT reconstructions, magnified 3D CT renderings, and, when available, ventriculographic correlation may better define the number of pouches, their orifices, their wall continuity, and their relationship with calcific or thrombotic components.

Retrospective review of additional CT reconstructions partially supported this interpretation, as supplementary coronal and oblique views suggested a more complex basal/lateral wall morphology than that shown by the initially selected standard CT section. Nevertheless, these images were reconstructed from the original preoperative dataset and were not prospectively acquired as lesion-targeted off-axis imaging. Therefore, although they strengthen the suspicion of adjacent or partially overlapping pouch-like structures, they do not provide definitive anatomical resolution.

### 3.3. Role of Suspected Mural Thrombus in the Differential Diagnosis

The suspected mural thrombotic component deserves specific consideration. In patients with ischemic remodeling, regional akinesia, scar, and altered ventricular geometry may favor thrombus formation, particularly adjacent to chronically abnormal myocardial segments. At the same time, thrombotic material may complicate interpretation by partially filling an outpouching, mimicking an intracardiac mass, or obscuring the true relationship between the lesion and the ventricular wall. Therefore, the differential diagnosis in the present case was not limited to pseudoaneurysm versus diverticulum, but also included the possibility of thrombus associated with chronic post-ischemic remodeling or a thrombotic component superimposed on a structural lesion [5].

A standardized echocardiographic approach is important in this setting. Morphology, site of attachment, mobility, echogenicity, borders, and relationship with the ventricular wall should be systematically assessed, as these features may help differentiate thrombus from other intracardiac masses and guide the need for further imaging. In the present case, transthoracic echocardiography identified the lateral left ventricular outpouching and regional wall motion abnormalities, but did not definitively characterize a thrombotic component. Cardiac magnetic resonance provided the strongest support for this possibility, showing a hypointense component compatible with thrombus in relation to transmural scar. However, because no pathological confirmation was obtained, the finding is best described as suspected mural thrombus or imaging findings compatible with thrombus rather than definite thrombosis.

### 3.4. Why Surgery Reopened the Differential Diagnosis

The operative finding of two calcified posterolateral formations substantially broadened the diagnostic field. A chronic calcified pseudoaneurysm remains possible, but the presence of two small formations and the apparent continuity of their external wall with the ventricular wall argue against a straightforward pseudoaneurysm interpretation. In the clinical context of chronic coronary syndrome and regional ischemic remodeling, small chronic aneurysmal formations may represent an equally plausible, and perhaps more anatomically consistent, explanation. A diverticular process also remains conceivable, especially given the chronic, apparently stable, fibro-calcific appearance, although the ischemic substrate, wall-motion abnormalities, and scar burden make a purely congenital diverticulum less persuasive [10].

Notably, preoperative imaging localized the lesion predominantly to the basal lateral or anterolateral wall, whereas surgery identified two calcified formations on the posterolateral aspect of the ventricle. This discrepancy raises the possibility that the different modalities captured different components of a more complex chronic lesion rather than the same structure in identical terms.

A third possibility is that the lesion represents chronic post-infarction remodeling with fibro-calcific sacculations or contained cavities that do not fit neatly within classical categories. In that sense, the discrepancy may stem not from incorrect imaging, but from the limitations of how such lesions are currently classified.

### 3.5. A Gray-Zone Lesion Rather than a Forced Diagnosis

The most important conceptual point is that this lesion may belong to a diagnostic gray zone between small chronic aneurysmal formations, pseudoaneurysm, diverticulum, and chronic fibro-calcific post-infarction remodeling. The purpose of the report is not to impose a label unsupported by pathology, but to show that such gray-zone lesions exist and may challenge even extensive imaging work-up.

For that reason, the unresolved nature of the case is not a weakness to conceal but a finding to emphasize. The intraoperative shift from a presumed pseudoaneurysm to surgically described fibro-calcific diverticula is itself clinically informative.

### 3.6. Surgical Management

No direct surgical intervention was performed on the formations because of their posterolateral location, difficult exposure, chronic calcified appearance, and the high incremental risk of repair or resection. The lesions were not perceived intraoperatively as carrying immediate rupture risk. In this setting, limiting the operation to myocardial revascularization was a pragmatic and defensible strategy [4].

### 3.7. Limitations

No tissue diagnosis was obtained because the lesions were neither resected nor biopsied. Consequently, the suspected mural thrombotic component suggested by CMR could not be confirmed histologically, and its exact relationship with the fibro-calcific formations observed intraoperatively remains uncertain. Accordingly, the final classification remains inferential. In addition, the exact three-dimensional relationship between the imaging findings and the operative findings cannot be reconstructed with certainty. As with all single-case reports, the observations are hypothesis-generating rather than definitive.

Another important limitation is that the available imaging protocol was not specifically tailored to the possibility of two adjacent outpouching-like structures. Standard CT and CMR planes depicted a dominant cavity but did not reliably separate the suspected second pouch or define its orifice. Dedicated off-axis CMR sections, additional cine and late gadolinium enhancement planes targeted to the lesion, multiplanar CT reconstructions, and greater magnification of the 3D CT reconstruction might have provided more precise anatomical information. Similarly, color Doppler echocardiography focused on both suspected pouches could have helped demonstrate whether flow was present in one or both cavities. A ventriculographic image showing contrast opacification of the lateral wall lesion would also have been useful to clarify whether one or two pouches communicated with the left ventricular cavity. Therefore, the exact size, neck morphology, and communication of the suspected second pouch remain uncertain.

Although additional CT reconstructions were reviewed and added as Supplementary Material, they were obtained from the original preoperative CT dataset and not from a dedicated postoperative or prospectively lesion-targeted imaging protocol. Therefore, they could improve the visual appreciation of the complex lateral wall morphology but could not definitively determine the number of cavities, the individual neck or orifice of each suspected pouch-like component, or the presence of flow within each structure. Dedicated postoperative imaging, ideally focused echocardiography with color Doppler, contrast echocardiography where available, or repeat CT/CMR with lesion-targeted off-axis planes, would be required for definitive anatomical clarification.

## 4. Conclusions

This case illustrates that even comprehensive imaging may overstate diagnostic certainty in unusual chronic left ventricular lesions. In a patient with multivessel coronary artery disease, multimodality imaging consistently suggested a left ventricular pseudoaneurysm, whereas surgery revealed two calcified posterolateral formations with a markedly different gross appearance.

Whether these structures represented small chronic aneurysmal formations, an unusual chronic pseudoaneurysm, a diverticular process, chronic fibro-calcific post-infarction remodeling, or a mixed lesion cannot be resolved definitively. Similarly, the suspected thrombotic component should be interpreted cautiously as an imaging-based finding rather than a pathologically confirmed diagnosis. The case also illustrates that standard imaging planes may be insufficient when adjacent or partially overlapping ventricular wall outpouchings are present, and that lesion-targeted off-axis imaging may be necessary to clarify unexpected anatomy.

That uncertainty is precisely the message. Multimodality agreement does not necessarily equal diagnostic truth, and operative findings may still redefine the lesion in fundamental ways. Cases such as this deserve to be reported not despite their ambiguity, but because of it.

Additional retrospective CT reconstructions supported the impression of complex, possibly overlapping lateral wall outpouching-like components, but definitive anatomical classification would require dedicated lesion-targeted follow-up imaging.

## Figures and Tables

**Figure 1:**
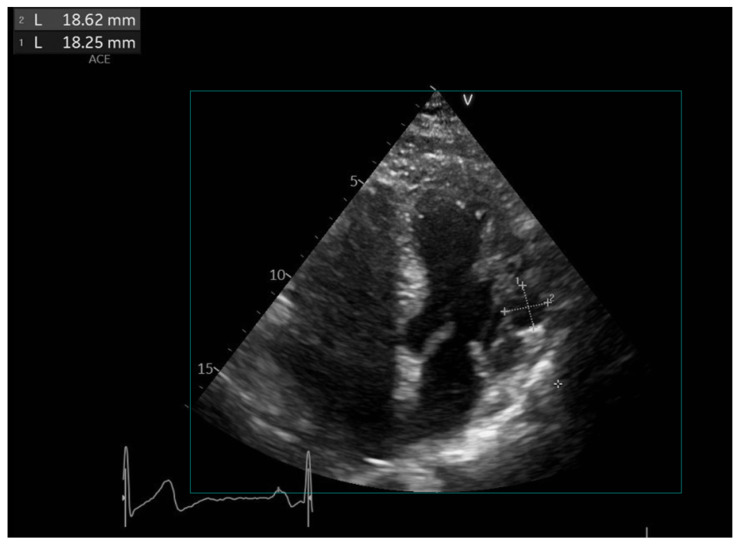
Preoperative transthoracic echocardiography, apical four-chamber view. The image demonstrates a basal lateral left ventricular outpouching-like structure, with the superior component measured at approximately 18.3 × 18.6 mm. Retrospective review suggests the possible presence of a second adjacent inferior pouch-like space, located below the posterior mitral leaflet, separated from the measured cavity by a hyperechogenic structure possibly corresponding to calcification. The precise number, morphology, and communication of these cavities with the left ventricular lumen could not be definitively established by standard transthoracic echocardiography alone.

**Figure 2:**
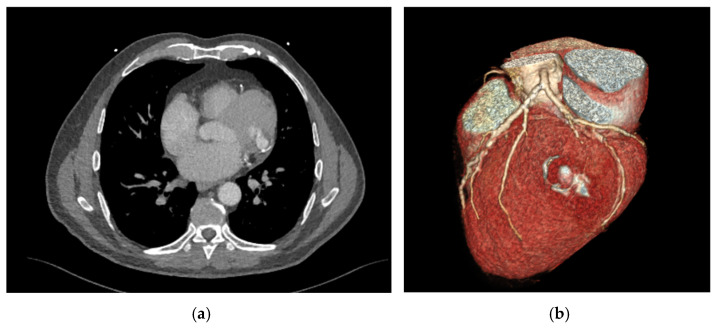
Contrast-enhanced cardiac computed tomography. (**a**) Standard axial section showing a focal lateral left ventricular outpouching-like abnormality with contrast opacification synchronous with the left ventricular cavity. In this plane, the lesion appears predominantly as a single cavity, and the relationship between possible adjacent pouch-like components cannot be reliably separated. (**b**) Three-dimensional volume-rendered cardiac CT reconstruction showing a more complex lateral wall protrusion. Retrospective review suggests the possible presence of two adjacent or partially overlapping pouch-like structures. Greater magnification and lesion-targeted reconstructions may have provided more precise anatomical definition.

**Figure 3:**
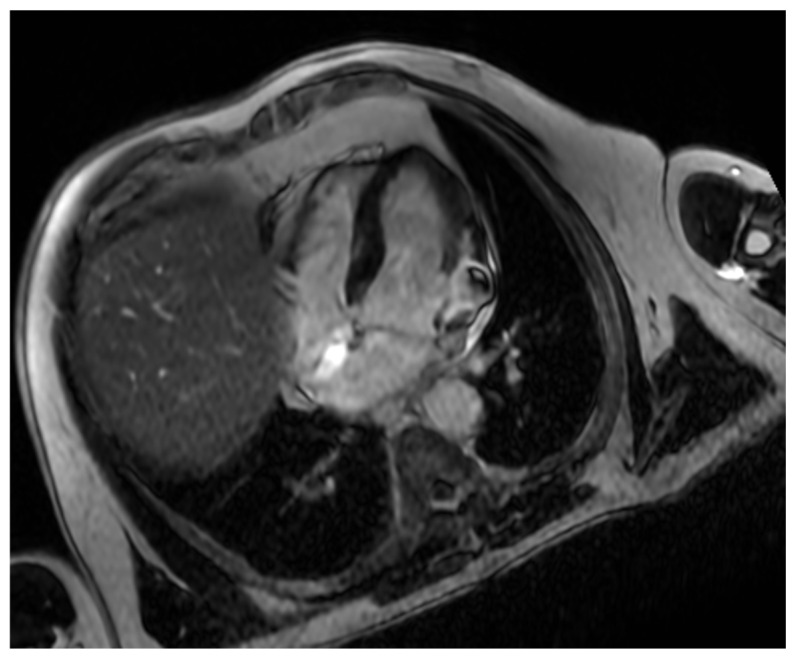
Preoperative cardiac magnetic resonance image showing a dominant focal lateral left ventricular outpouching with adjacent myocardial alteration in the medio-basal wall. In this standard imaging plane, the lesion appears predominantly as a single outpouching, contributing to the preoperative impression of chronic pseudoaneurysm. However, this section may not fully characterize the complex three-dimensional morphology suggested by echocardiography, 3D CT reconstruction, and intraoperative inspection.

**Figure 4:**
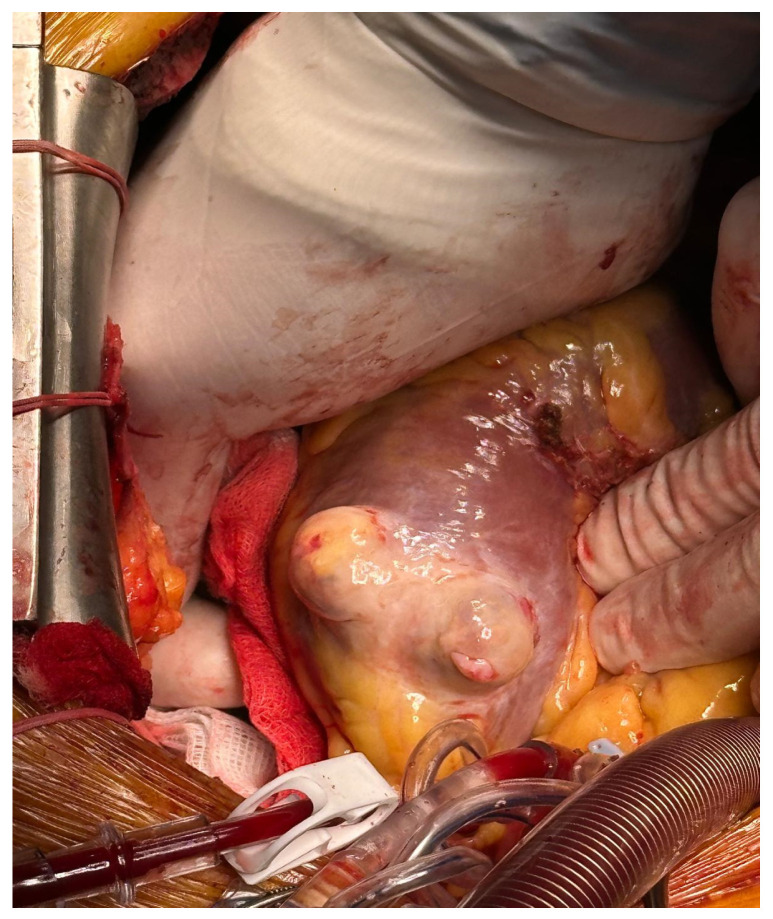
Intraoperative surgical image of the posterolateral aspect of the left ventricle. Two small fibro-calcific outpouching-like formations are visible in a surgically difficult-to-access region. Their external wall appeared grossly continuous with the ventricular wall, a feature that broadened the differential diagnosis toward small chronic aneurysmal formations, calcified diverticular structures, or chronic post-ischemic fibro-calcific remodeling rather than a straightforward pseudoaneurysm. Because of their chronic calcified appearance, difficult exposure, and the high risk of additional manipulation, no direct surgical intervention was performed on these structures.

**Table 1:** Clinical timeline of the patient.

Date/Interval	Clinical Events and Findings
Months before admission	Effort-related chest pain, occasional chest discomfort at rest, exertional dyspnea, fatigability
Initial cardiology work-up (November 2025)	CT suggested basal anterolateral LV pseudoaneurysm (3.2 × 2.4 × 1.9 cm); TTE showed preserved LVEF (~50%) with regional wall motion abnormalities and suspected pseudoaneurysm
Coronary angiography/ventriculography	Severe multivessel CAD; communication between LV cavity and lateral wall lesion (~2.0 × 2.5 cm), without pericardial extravasation
Preoperative reassessment (January 2026)	TTE again favoured lateral wall pseudoaneurysm
Cardiac magnetic resonance (27 January 2026)	Narrow-neck LV pseudoaneurysm (20 × 23 mm; neck ~7 mm), suspected mural thrombus, transmural fibrosis of the medio-basal lateral wall, additional inferior/inferoseptal ischemic scar
Surgery (6 February 2026)	Intraoperative discovery of two posterolateral LV fibro-calcific formations, discordant with imaging; CABG performed without direct intervention on the lesion
Postoperative period	Favourable recovery; postoperative TTE with LVEF ~50%; discharge in stable condition

## Data Availability

The original contributions presented in this study are included in the article. Further inquiries can be directed to the corresponding authors.

## Supplementary Materials

The following supporting information can be downloaded at: https://www.mdpi.com/article/10.3390/life16060908/s1. Supplementary Figure S1. Additional coronal and oblique CT reconstructions showing a basal/lateral left ventricular outpouching-like abnormality with possible adjacent or partially overlapping pouch-like components, with a maximal measured dimension of approximately 3.08 cm. The available CT dataset did not allow definitive separation of the individual orifice of each suspected component.

## Abbreviations

The following abbreviations are used in this manuscript:

bpm	beats per minute
CABG	coronary artery bypass grafting
CAD	coronary artery disease
CT	computed tomography
LAD	left anterior descending artery
LV	left ventricle
LVEF	left ventricular ejection fraction
OM	obtuse marginal branch
TTE	transthoracic echocardiography
