# Supplementary material for: Aneurysm, Pseudoaneurysm, Diverticulum, or Other? Discordance Between Multimodality Imaging and Surgical Findings in a Patient with Coronary Artery Disease
Source: Life (Basel). 2026 May 28;16(6):908. doi: 10.3390/life16060908 (PMC13302019; doi:10.3390/life16060908)
Supplement: Supplementary file 1 [file life-16-00908-s001.zip › life-4313198-supplementary.pdf]

CARE case report guidelines

CARE Checklist of information to include when writing a case report

(cc) BY-NC-ND

Topic	Item	Checklist item description
Title	1	**Pseudoaneurysm, Diverticulum, or neither? Discordance between multimodality imaging and surgical findings in a patient with coronary artery disease**
Key Words	2	left ventricular pseudoaneurysm; multimodality imaging; case report; coronary artery bypass grafting; diagnostic discordance.
Abstract	3a	Introduction: Left ventricular outpouchings are difficult to classify; multimodality imaging may overestimate diagnostic certainty in complex chronic lesions..
	3b	Main clinical features: 66-year-old male with chronic coronary syndrome, multivessel CAD, and imaging findings suggesting LV pseudoaneurysm
	3c	The main diagnoses, therapeutic interventions, and outcomes: Suspected LV pseudoaneurysm on all imaging modalities; intraoperatively two fibro-calcific formations identified instead; CABG performed; favorable postoperative outcome.
	3d	Conclusion— Multimodality imaging may not reflect true anatomy; diagnostic uncertainty must be acknowledged in complex LV lesions..
Introduction	4	LV outpouchings include aneurysm, pseudoaneurysm, diverticulum, and remodeling patterns; differentiation is clinically important but often difficult despite advanced imaging..
Patient Information	5a	De-identified patient specific information: 66-year-old male with chronic coronary syndrome.
	5b	Primary concerns and symptoms of the patient: Chest pain, dyspnea, fatigability.
	5c	Medical: Hypertension, dyslipidemia, smoking, CKD, overweight, heart failure (NYHA II)._
	5d	Relevant past interventions with outcomes: No major prior cardiac surgery reported; diagnostic cardiology work-up performed.
Clinical Findings	6	Describe significant physical examination (PE) and important clinical findings: On clinical and paraclinical evaluation, the patient presented with sinus rhythm and electrocardiographic changes consisting of negative T waves in the lateral leads. Transthoracic echocardiography revealed a non-dilated left ventricle with a preserved ejection fraction of approximately 50%, associated with regional wall motion abnormalities involving the posterior, inferior, and lateral walls. Additionally, a basal lateral left ventricular outpouching was identified, raising suspicion for a structural ventricular lesion.
Timeline	7	Historical and current information from this episode of care organized as a timeline: The patient reported several months of progressive effort-related chest pain, occasionally present at rest, associated with exertional dyspnea and fatigability. Initial cardiologic evaluation in November 2025 identified severe multivessel coronary artery disease and a suspected left ventricular pseudoaneurysm on imaging. Subsequent investigations, including echocardiography, computed tomography, ventriculography, and cardiac magnetic resonance imaging in January 2026, consistently supported the diagnosis of a left ventricular pseudoaneurysm. The patient underwent surgical intervention in February 2026, during which coronary artery bypass grafting was performed. Intraoperatively, the suspected pseudoaneurysm was not confirmed, and two fibro-calcific formations were identified instead. The postoperative course was favorable, and the patient was discharged in stable condition

Diagnostic Assessment

8a Diagnostic testing (such as PE, laboratory testing, imaging, surveys): Diagnostic evaluation included electrocardiography, which demonstrated sinus rhythm and lateral repolarization abnormalities. Transthoracic echocardiography revealed preserved left ventricular ejection fraction with regional wall motion abnormalities and a basal lateral outpouching suggestive of pseudoaneurysm. Coronary angiography confirmed severe multivessel coronary artery disease. Left ventriculography demonstrated a communication between the left ventricular cavity and the lateral wall lesion. Contrast-enhanced computed tomography showed a well-defined aneurysmal cavity communicating with the ventricular lumen. Cardiac magnetic resonance imaging further supported the diagnosis by demonstrating a narrow-neck outpouching, mural thrombus, and transmural fibrosis in the affected region..

8b Diagnostic challenges: Despite concordant findings across multiple imaging modalities, the true anatomical nature of the lesion remained uncertain. The imaging studies strongly suggested a left ventricular pseudoaneurysm; however, intraoperative findings revealed structures that did not correlate with the expected morphology. This discrepancy highlights the limitations of imaging in differentiating complex chronic ventricular lesions.

8c Diagnosis: The preoperative diagnosis was a chronic left ventricular pseudoaneurysm associated with multivessel coronary artery disease. Following surgical exploration, the diagnosis remained inconclusive, as the lesion did not correspond to a typical pseudoaneurysm and was instead described as fibro-calcific formations of uncertain nature.

8d Prognosis: Given the presence of severe multivessel coronary artery disease and left ventricular dysfunction, the patient was considered at moderate surgical risk. The postoperative evolution was favorable, with stable cardiac function and no immediate complications..

Therapeutic Intervention

9a Types of therapeutic intervention: The patient underwent surgical myocardial revascularization through coronary artery bypass grafting..

9b Administration of therapeutic intervention: The procedure consisted of a left internal mammary artery graft to the left anterior descending artery and an autologous saphenous vein graft to the obtuse marginal branch. The operation was performed under standard cardiopulmonary bypass conditions.

9c Changes in therapeutic intervention: No direct surgical intervention was performed on the left ventricular lesion due to its difficult anatomical location, calcified appearance, and the increased operative risk associated with further manipulation..

Follow-up and Outcomes

10a Clinician and patient-assessed outcomes: The postoperative course was favorable, with the patient remaining hemodynamically stable and showing no signs of clinical deterioration.

10b Important follow-up diagnostic and other test results: Follow-up transthoracic echocardiography demonstrated a non-dilated left ventricle with preserved ejection fraction of approximately 50%, persistent regional wall motion abnormalities, and no evidence of pericardial or pleural effusion.

10c Intervention adherence and tolerability: The surgical intervention was well tolerated, with no immediate postoperative complications requiring additional intervention

10d Adverse and unanticipated events: No significant adverse or unanticipated postoperative events were reported.

Discussion

11a A scientific discussion of the strengths and limitations: This case is strengthened by the comprehensive use of multimodality imaging and the direct comparison with intraoperative findings. However, it is limited by the absence of histopathological confirmation and the inability to establish a definitive diagnosis.

11b Discussion of the relevant medical literature**:** The findings are consistent with existing literature emphasizing the complexity of differentiating left ventricular outpouchings and the limitations of imaging modalities in chronic ischemic lesions.

11c The scientific rationale for any conclusions: Chronic ischemic remodeling, fibrosis, calcification, and thrombus formation can produce complex ventricular structures that mimic classical entities such as pseudoaneurysms or diverticula, leading to diagnostic uncertainty even with advanced imaging

11d The primary "take-away" lessons of this case report: This case demonstrates that concordant multimodality imaging does not necessarily guarantee diagnostic accuracy, and surgical findings may significantly alter the understanding of the lesion. Diagnostic caution and acknowledgment of gray-zone entities are essential.

Patient Perspective

12 The patient should share their perspective: A patient perspective was not included in this report..

Informed Consent **13** Did the patient give informed consent?: **Yes** ☑ **No**☐ Written informed consent for publication was obtained from the patient.
