# Supplementary figures and images for: Aneurysm, Pseudoaneurysm, Diverticulum, or Other? Discordance Between Multimodality Imaging and Surgical Findings in a Patient with Coronary Artery Disease
Source: Life (Basel). 2026 May 28;16(6):908. doi: 10.3390/life16060908 (PMC13302019; doi:10.3390/life16060908)

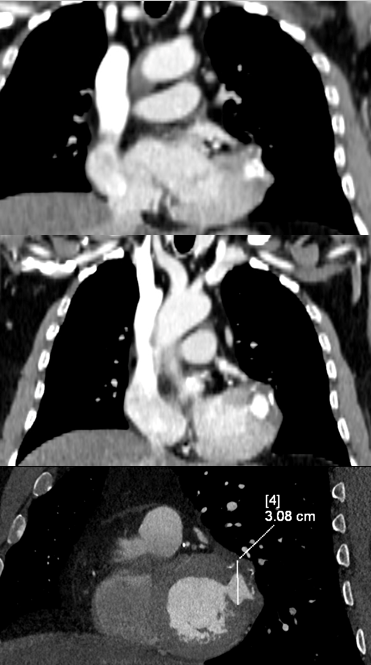

Supplement: Supplementary file 1 [file life-16-00908-s001.zip › life-4313198-supplementary figure S1.png]
